# Integrins and NAFLD-associated liver diseases: clinical associations, pathophysiological mechanisms and pharmacological implications: Integrins and NAFLD-associated liver diseases

**DOI:** 10.3724/abbs.2024149

**Published:** 2024-09-14

**Authors:** Yangyue Ni, Mengwen Huang, Shiyang Chen, Shihui Wang, Jianfeng Chen

**Affiliations:** 1 Key Laboratory of Systems Health Science of Zhejiang Province School of Life Science Hangzhou Institute for Advanced Study University of Chinese Academy of Sciences Hangzhou 310024 China; 2 Key Laboratory of Multi-Cell Systems Shanghai Institute of Biochemistry and Cell Biology Center for Excellence in Molecular Cell Science Chinese Academy of Sciences University of Chinese Academy of Sciences Shanghai 200031 China

**Keywords:** integrins, NAFLD, NASH, NAFLD-related HCC, diagnostics and treatments

## Abstract

Nonalcoholic fatty liver disease (NAFLD) is a leading cause of chronic liver disease and poses a substantial health burden with increasing incidence globally. NAFLD encompasses a spectrum extending from hepatic steatosis to nonalcoholic steatohepatitis (NASH), with the possibility of progressing to cirrhosis or, in severe instances, hepatocellular carcinoma (HCC). NAFLD extends beyond simple metabolic disruption and involves multiple immune cell-mediated inflammatory processes. Integrins are a family of heterodimeric transmembrane cell adhesion receptors that regulate various aspects of NAFLD onset and progression, including hepatocellular steatosis, hepatic stellate cell (HSC) activation and immune cell infiltration. In this review, we comprehensively summarize the involvement of integrins in NAFLD, as well as the downstream signal transduction mediated by these receptors. Furthermore, we present the latest clinical and preclinical findings on drugs that target integrins for steatosis, inflammation, fibrosis and NAFLD-related HCC treatment.

## Introduction

Liver disease is a worldwide health care concern, contributing to more than two million deaths annually and accounting for 4% of all deaths worldwide [1]. At present, nonalcoholic fatty liver disease (NAFLD) is the predominant etiology of chronic hepatitis in Western societies, with its prevalence rapidly increasing on a global scale [2]. Notably, this condition impacts a quarter of the global adult population. Over the past two decades, with rapid lifestyle changes, NAFLD has become the most common liver disease in China. The prevalence of NAFLD has increased from 23.8% to 29.0%; however, NAFLD has not received sufficient attention [3]. Over the past 30 years, the total number of deaths among NAFLD patients worldwide has doubled. The overall percentage of deaths attributed to NAFLD-related causes has increased from 0.10% to 0.17% [1]. Owing to continued high rates of adult obesity and type 2 diabetes mellitus, coupled with an aging population, the total NAFLD population is forecasted to increase by 18.3% in the U.S., whereas the prevalence of nonalcoholic steatohepatitis (NASH) cases is expected to increase by 56% by 2030 [ 4, 5]. Currently, NAFL is defined as a catch-all term that encompasses a variety of disorders characterized by steatosis affecting a minimum of 5% of hepatocytes, combined with metabolic risk factors (such as type 2 diabetes and obesity). This definition excludes heavy alcohol usage-induced hepatitis or other chronic liver diseases. Furthermore, NASH is characterized by hepatic damage, including hepatocyte ballooning degeneration, diffuse lobular inflammation, and fibrosis [6]. Up to 15% of individuals with NAFLD progress to NASH, the more severe form of the disease. Whereas simple steatosis is often considered a “benign” condition, individuals with NASH face the potential for progression to fibrosis, cirrhosis and hepatocellular carcinoma (HCC) and have an elevated risk of liver-related mortality. In addition, HCC may develop in individuals with NAFLD without cirrhosis, termed NAFLD-HCC [7].

**Figure 1:**
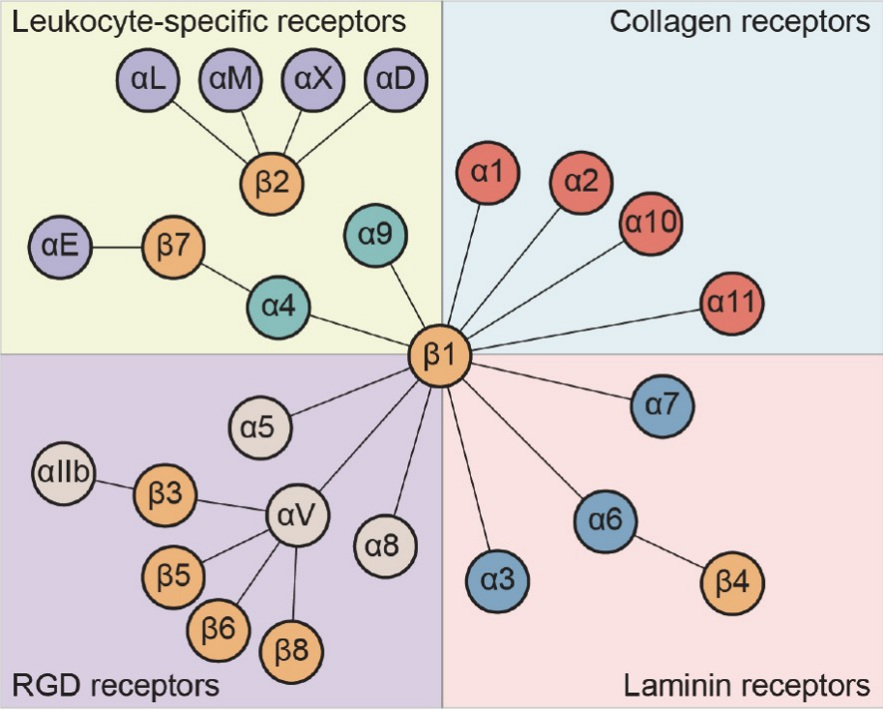
Figure 1 Integrin family and classification Twenty four integrins consist of 18 α subunits and 8 β subunits, which can be classified into RGD-binding integrins, leukocyte cell-adhesion integrins, collagen-binding integrins, and laminin-binding integrins.

Integrins are α/β heterodimeric cell adhesion molecules, mediating cell-cell, cell-extracellular matrix (ECM) and cell-pathogen interactions and transmit signals bidirectionally across the plasma membrane [8]. In vertebrates, 18 α subunits and 8 β subunits exist, which combine into 24 types of integrins that are broadly distributed across numerous organs and tissues ( Figure 1). Integrins, as type I transmembrane proteins, control cell-cell and cell-ECM adhesion, thereby impacting a variety of cellular functions, including migration, proliferation, wound repair, and other cellular activities [9]. In NAFLD, integrins serve as the primary mechanism by which cells in the liver sense their extracellular environment, such as accumulated lipids that trigger the “first hit” in NAFLD, which involves insulin resistance (IR) and hepatic steatosis [10]. Liver tissue eventually undergoes lipid peroxidation, endoplasmic reticulum (ER) stress, oxidative stress, inflammatory damage, and other pathological alterations, contributing to the development of the “multiple-hit” scenario [11]. During NAFLD-induced fibrogenesis, integrins mediate diverse cell-matrix and cell-cell interactions. In NASH-related HCC, which progresses annually [12], integrins are typically dysregulated and are involved in nearly every stage of cancer progression, including epithelial-mesenchymal transition (EMT), angiogenesis, cell proliferation, adhesion, and invasion [13]. Moreover, integrins are considered to influence multiple components within the tumor microenvironment of HCC, such as lymphocyte activation, migration, and extravasation.

Given the lack of Food and Drug Administration (FDA)-approved medications for the treatment of NAFLD, the pharmacological interventions available for NAFLD are currently restricted. NAFLD and NASH are growing worldwide health concerns and significant unmet medical requirements [14]. The redundancy of integrin functions and their distinct roles at different stages of NAFLD highlight the considerable therapeutic potential of these molecules. This review focuses on the aberrant expression, activation, and signaling of integrins in both liver parenchymal and nonparenchymal cells, providing a summary of current findings regarding the involvement of integrins in NAFLD. Furthermore, we summarize the present state and treatment approaches of anti-integrin medications in both preclinical and clinical practice, encompassing a broad spectrum from NAFLD to HCC.

## The Structure of Integrins

**Figure 2:**
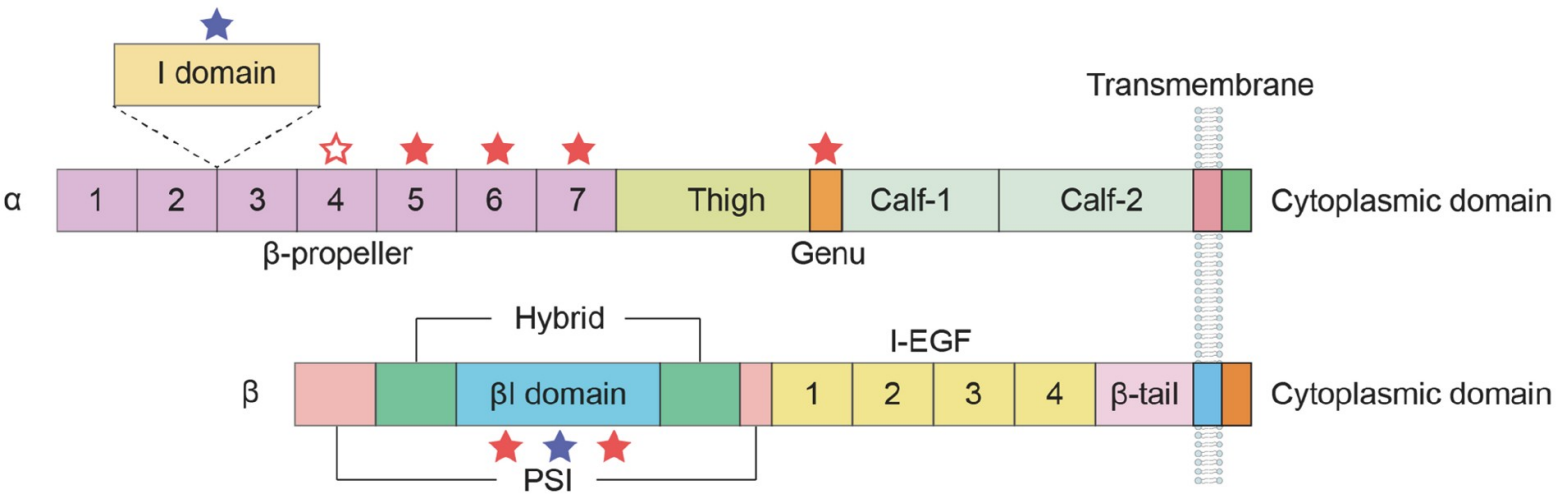
Figure 2 Representation of the structure of the integrin α and β subunits Integrins are composed of α and β subunits, forming heterodimeric transmembrane glycoproteins. The α-chain consists of four or five extracellular domains: a seven-bladed β-propeller, a thigh, and two calf domains. Nine of the 18 integrin α chains contain an αI domain. The β subunit comprises seven domains with flexible and complex interconnections. The red and blue asterisks denote Ca2+- and Mg2+-binding sites, respectively. The hollow asterisk denotes the Ca2+-binding site in the fourth repeat of the β-propeller domain in certain α subunits.

Integrins are α/β heterodimeric glycoprotein receptors [ 15, 16]. The α subunit comprises a seven-bladed β-propeller linked to a thigh, a calf-1 and a calf-2 domain, creating the leg structure that provides support for the integrin head. Seven repetitive motifs constitute the shared structure among various α subunits within their extracellular domains, developing a seven-bladed propeller structure on the upper surface. The ectodomain of the β subunit is composed of seven domains with intricate domain insertions: a plexin-semaphorin-integrin (PSI) domain, a βI domain inserted in the hybrid domain, four cysteine-rich epidermal growth factor (EGF) modules, and a β-tail domain (βTD) domain [17]. Integrins can be classified into two subfamilies on the basis of the presence of the αI domain. The αI domain, also known as the αA domain, is present in nine of the integrin α subunits (α1, α2, α10, α11, αD, αE, αX, αM and αL). This domain is inserted between blades two and three of the β-propeller structure. In integrins containing αI, the α head consists of β-propeller and αI domains, whereas in integrins lacking αI, a single β-propeller forms the α head [18]. αI domain-containing integrins bind to ligands via the αI domain [19]. The metal ion-dependent adhesion site (MIDAS), located on the top surface of the αI domain, is essential for the interaction between ligands and integrins. The βI domain shares a similar Rossmann fold with the αI domain, featuring a central six-stranded β-sheet surrounded by eight helices. In αI-less integrins, the MIDAS site in the βI domain mediates ligand binding ( Figure 2).

Typically, integrins bind to their ligands by recognizing short, acidic peptide motifs (such as RGD and LDV), which are conserved tripeptide sequences. To date, two β1 integrins (α5 and α8), all five αV integrins, and αIIbβ3 are capable of binding to the Arg-Gly-Asp (RGD) sequence. This tripeptide is present in vitronectin, fibronectin, osteopontin, fibrinogen, collagen, thrombospondin, and von Willebrand factor [20]. Integrins αEβ7, α4β7, α4β1, and α9β1 bind to the specific Leu/Ile-Asp/Glu-Val/Ser/Thr (LDV) motif. The binding sites within the ligands of β2 integrins are structurally similar to the LDV motif [21]. Additionally, certain integrins are also capable of identifying the triple-helical GFOGER sequence. Notably, integrin αVβ3 is highly expressed in activated HSCs and promotes the survival and proliferation of HSCs during liver fibrosis. With the activation of HSCs, integrin αVβ3 expressed on HSCs binds to the RGD motif in various ECM components [22].

## Activation and Signal Transmission of Integrins

Integrin activation is tightly regulated and is essential for cellular functions. The activation of integrins occurs through intricate signaling mechanisms both inside and outside the cells, leading to conformational changes in the integrin molecules. These changes expose the ligand-binding site in integrins, allowing them to interact with specific ligands present in the ECM or on the surface of neighboring cells.

**Figure 3:**
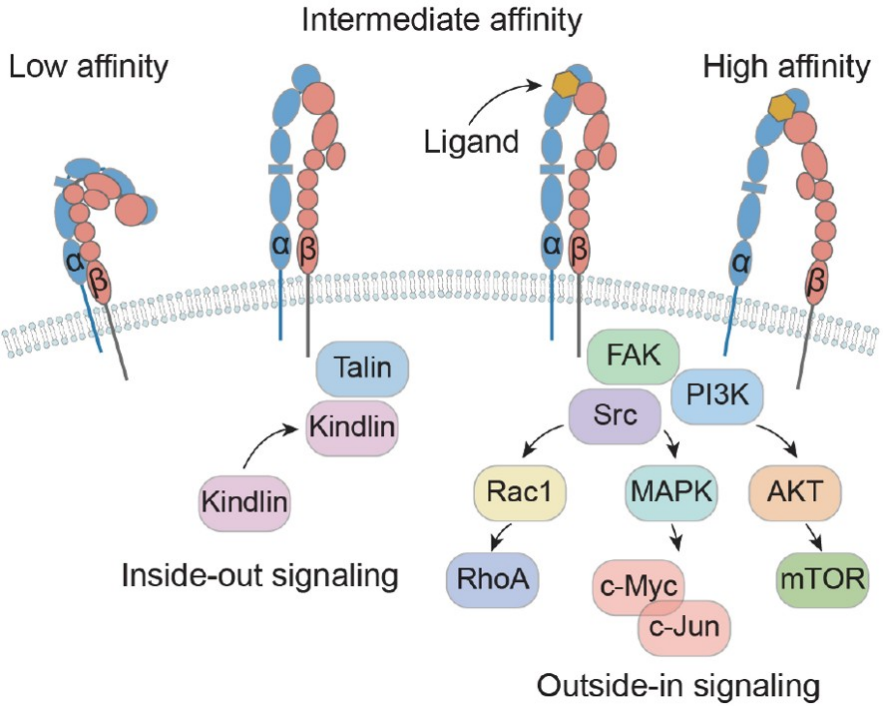
Figure 3 Activation and signaling of integrins Integrins can adopt at least three distinct conformational states, each with varying affinities for ligands: the low-affinity “bent” conformation, the intermediate-affinity “extended conformation with closed headpiece”, and the high-affinity “extended conformation with open headpiece”. Integrins mediate cell signaling transduction through two mechanisms, known as “inside-out” signaling and “outside-in” signaling. In the “inside-out” signaling pathway, intracellular signals induce conformational changes in integrins, altering their ligand-binding affinity. Conversely, in the “outside-in” signaling pathway, engagement with extracellular ligands triggers conformational changes in integrins, transmitting signals into the cell and initiating downstream signaling cascades.

Integrin activation involves intricate and reversible conformational changes within these transmembrane receptors. In the low-affinity state, integrins maintain a bent V-shaped conformation, wherein the head is positioned in the membrane-proximal regions of the legs. This conformation is sustained by the α/β salt bridge in the inner membrane region and helix packing of the transmembrane region. Upon activation, the head of the integrin extends, exposing the ligand-binding site, while the intracellular tails of the integrin separate [23]. Integrins are capable of adopting at least three primary distinct conformational states, each with different affinities for ligands: the low-affinity “bent” conformation, the intermediate-affinity “extended conformation with a closed headpiece”, and the high-affinity “extended conformation with an open headpiece” [24]. The delicate balance between these conformations has a substantial effect on controlling both the affinities for cell adhesion and the intensity of communication. Integrins can initiate “inside-out” and “outside-in” bidirectional signaling, rapidly resulting in global conformational rearrangement. Within the “inside-out” signaling pathway, intracellular activators such as talin or kindlin attach to the cytoplasmic tail of integrin β subunits [25]. This interaction triggers integrins to undergo conformational changes from a low-affinity bent shape to a high-affinity extended conformation, which recruits multivalent protein complexes that cluster together and strengthen their affinity for ligands. Consequently, this biological process facilitates essential cellular activities, such as cell adhesion, cell migration, ECM assembly and remodeling. In contrast, in “outside-in” signaling, integrin receptors engage with external ligands, such as ECM components, growth factor receptors (GFRs), urokinase plasminogen activator receptors, and transforming growth factor-β (TGF-β) receptors [26]. Binding of these ligands to integrin extracellular domains leads to integrin clustering and the transmission of signals into the cellular interior. This signaling cascade subsequently instigates alterations in cell polarity, cytoskeletal structure, and gene transcription ( Figure 3).

## Integrins Guide the Trafficking of Immune Cells to the Liver

The endothelium acts as a barrier, separating circulating immune cells from inflamed tissues. Integrins are important in the process of immune cell trafficking, orchestrating a complex adhesion cascade that encompasses tethering and rolling of immune cells along the walls of high endothelial venules, chemokine-induced activation, firm arrest, and transendothelial migration [27]. Initially, immune cells undergo tethering and rolling, which is controlled by the interaction of selectins with their respective ligands. Leukocyte-expressed L-selectin (also known as CD62L) mediates tethering and rolling through recognition of its counterreceptor (peripheral node addressin, PNAd) on high endothelial venules. This step is reversible unless firm adhesion occurs [28]. The activation induced by chemokines is a crucial step in the transition from rolling to firm arrest. Chemokines rapidly activate integrins via an “inside-out” signaling network that controls the connection between the cytoplasmic domains of integrins and intracellular effector proteins ( *e.g.*, talin or kindlin) during this process. Upon the binding of effector proteins, integrins transition from an inactive bent conformation to their active form, which is distinguished by its extended form and strong affinity for ligands [ 29, 30]. Specific endothelial ligands, including intercellular adhesion molecule (ICAM)-1/2, vascular cell adhesion molecule (VCAM)-1, and mucosal vascular addressin cell adhesion molecule (MAdCAM)-1, interact with activated leukocyte integrins, primarily α4β1, α4β7, αLβ2, αMβ2, αXβ2, and αDβ2, to mediate the firm adhesion of immune cells. Platelet and endothelial cell adhesion molecule (PECAM)-1 and junctional adhesion molecule (JAM)-A/B/C regulate the final step of transmigration through interactions with leukocyte lymphocyte function-associated antigen (LFA)-1 (αLβ2), very late antigen (VLA)-4 (α4β1) and macrophage-1 antigen (Mac-1) (αMβ2) [31].

Integrin-mediated adhesion plays a crucial role in guiding lymphocyte localization toward the liver. The binding of VCAM-1 to VLA-4 is crucial for localizing TH1-type CD4 ^+^ T cells [32] and activated CD8 ^+^ T cells [33] in the liver. These adhesion molecules are involved in the antigen-independent homing of T cells to the liver, whereas ICAM-1 assumes a more critical role in antigen recognition by T cells. Effector CD8 ^+^ T cells traveling through the mouse liver initially halt in sinusoids, not postcapillary venules, independent of antigen recognition and a variety of molecules that are variably involved in leukocyte trafficking to different organs [34]. Conversely, the favored method for halting effector CD8 ^+^ T cells circulating in liver sinusoids involves docking onto platelets that have already bound to sinusoidal hyaluronan via CD44 [35]. Additional adhesion molecules are expressed in the hepatic vasculature during inflammation. MAdCAM-1 increases significantly in response to both IL-1β and TNF-α. Hepatic MAdCAM-1 interacts with integrin α4β7, which is typically expressed on gut-homing lymphocytes [36]. Furthermore, endothelial activation leads to increased expressions of various adhesion molecules, including ICAM-1, VCAM-1 and L-selectin [37]. Both VAP-1 and ICAM-1 play a role in Treg cell adhesion and transmigration [38].

## The Role of Integrins in the Development of Simple Steatosis in NAFLD

Integrins and cell adhesion molecules regulate a multitude of physiological and pathological processes by mediating the connections between cells and their external environment. Accumulating evidence highlights the crucial role of integrin-mediated signaling in various chronic and acute noncancerous diseases, with a particular emphasis on liver-related conditions. Integrins play pivotal roles in immune cells for trafficking, activation, and function to induce effective immune responses. During the progression from NAFLD to cirrhosis, integrins selectively manipulate specific subsets of immune cells to mediate pro- or anti-inflammatory pathological scenarios in the liver [ 39, 40]. Notably, integrins serve essential biological functions in hepatic nonimmune cells, mediating cell-matrix and cell-cell interactions [41].

**Figure 4:**
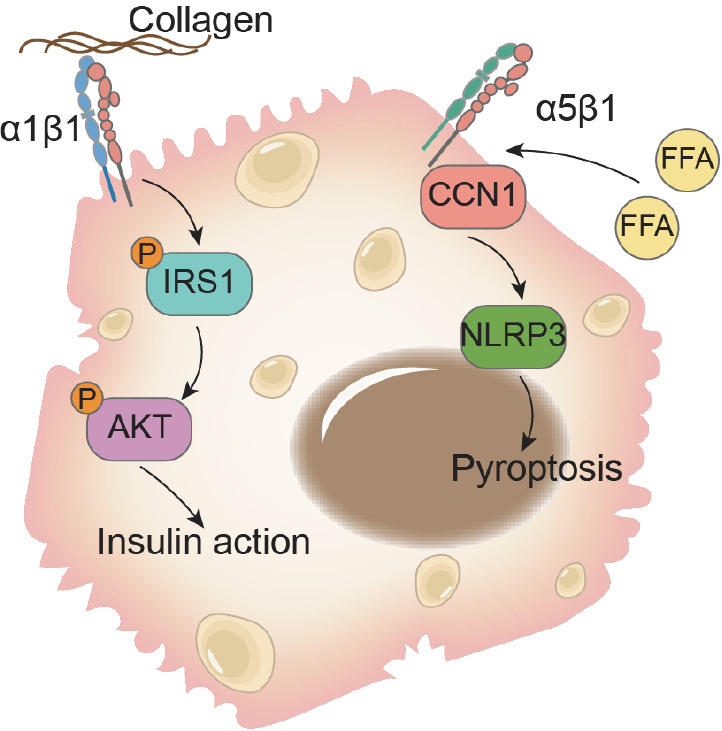
Figure 4 Roles of integrins in hepatocyte functions in NAFLD Hepatic integrin α1β1 induces the phosphorylation and subsequent activation of IRS1 and AKT, promoting liver insulin action and preventing diet-induced liver insulin resistance. Integrin α5β1 activates of NLRP3 inflammasome and pyroptosis in hepatocytes during NAFLD.

According to the most widespread and prevailing model of the “multiple-hit hypothesis”, the “first hit” involves liver lipid accumulation and insulin resistance [ 42, 43]. Hepatic lipid accumulation is associated with liver damage and an increase in the production of ECM [44]. α1β1, a collagen-binding integrin located on hepatocytes, provides protection against diet-induced hepatic insulin resistance while simultaneously promoting lipid accumulation in the liver [45]. Elevated circulating levels of free fatty acids (FFAs) strongly correlate with hepatic lipid accumulation in individuals with NAFLD. Experimental evidence has confirmed the involvement of integrin α5β1 in FFA-induced intracellular lipid accumulation, activation of the NLRP3 inflammasome, and proptosis in hepatocytes [46]. In contrast, hepatocyte-specific deletion of the integrin β1 subunit has been reported to alleviate hepatic insulin resistance in diet-induced obese mice, while liver triglyceride levels remain elevated ( Figure 4) [47].

## Integrins in the Development of NASH

Elevated levels of inflammatory cytokines, mitochondrial dysfunction, oxidative stress and adipokines are associated with the “second hit” of NAFLD, thereby driving the progression of the disease to hepatic steatosis and ultimately cirrhosis [48]. Triggers of hepatic inflammation contribute to the transition from NAFLD (isolated steatosis) to NASH [49]. Integrin and chemokine receptor pairs drive myeloid cell infiltration and residence in damaged tissues, thus creating a more intricate immune microenvironment [50]. Both recruited integrin αM ^+^ macrophages [51] and resident integrin αX ^+^ macrophages [52] are key factors in the development of simple steatosis to steatohepatitis. Type 1 conventional dendritic cells (cDC1s) have been identified as important drivers of liver pathology in NASH [53]. Integrin expression influences the heterogeneity of cDC1s. Integrin αE ^+^ cDC1s represent an anti-inflammatory subtype that protects the liver from metabolic damage during the development of steatohepatitis in mice [54].

The spectrum of hepatic lesions linked to NAFLD encompasses the infiltration and activation of adaptive immune cells, including T and B lymphocytes [55]. Evidence of ectopic expression of the gut-homing adhesion molecule integrin α4β7 was demonstrated in the hepatic T cells of NASH patients [56]. Although hepatic infiltrating β7-expressing T cells exhibit an aggravated proinflammatory phenotype, the role of integrin β7 in liver lipid accumulation and fibrotic pathology remains controversial. Research has shown that integrin β7 deficiency protects against atherosclerosis [57] and obesity-related insulin resistance [58], attenuating hepatic inflammation and fibrosis in NASH [39]. In contrast, gut-homing β7 ^+^ TH17 cells may be utilized to alleviate metabolic disorders and steatosis in obese individuals [59].

αV integrins regulate the activity of TGF-β, the master regulator of fibrosis, making them therapeutic targets. The hepatic sinusoid dominates lipid metabolism and tissue fibrosis. Laminin (LN), an integrin ECM ligand, is excessively deposited in gaps between liver sinusoidal endothelial cells (LSECs) in patients with NAFLD, reducing endothelial cell permeability and leading to sinusoidal capillarization. Damaged LSECs result in the expression of integrin αVβ3, subsequently inducing the expression of LN [60]. In addition, αVβ3 has been proposed as a central mediator of fibrosis in multiple organs and is highly expressed on activated hepatic stellate cells (HSCs) [61]. Integrin αVβ6 is markedly upregulated in hepatitis fibrosis, cirrhosis, and other liver injuries via the activation of TGF-β1 signaling in HSCs [62]. The integrins αVβ3 [ 63– 65], αVβ6 and αVβ8 [66] may have the potential to serve as markers and therapeutic targets for liver fibrosis. Unfortunately, no αV integrin inhibitors have reached the clinical market. Integrin α8β1 is selectively expressed in HSCs and is elevated in specimens from patients with liver fibrosis [67]. The administration of an anti-integrin α8 neutralizing monoclonal antibody improved pathology and fibrosis in cytotoxic (CCl _4_ treatment), cholestatic fibrosis and NASH-associated models [68].

**Figure 5:**
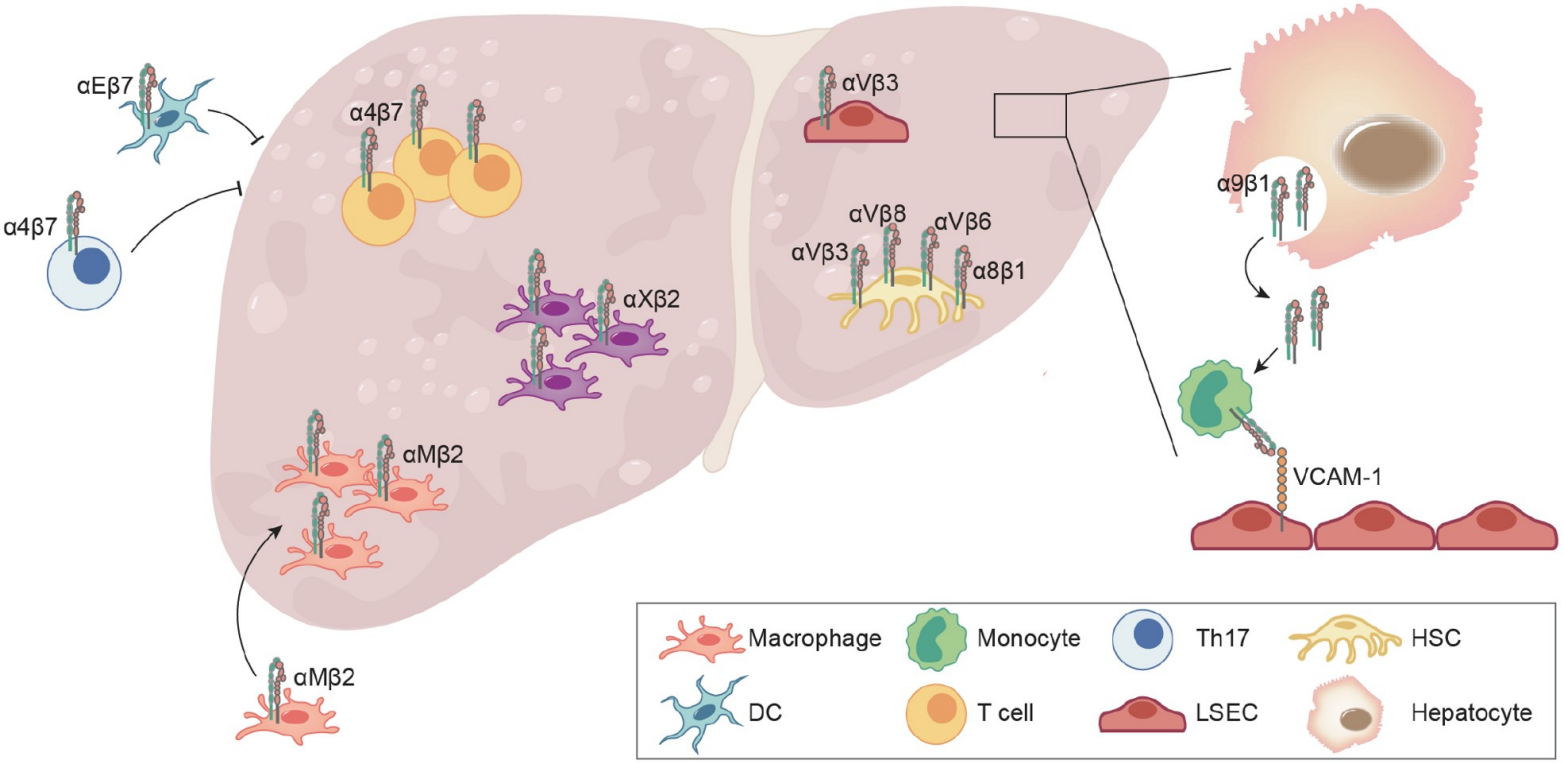
Figure 5 The role of integrins in liver fibrosis in NASH Recruited αM+ macrophages, αX+ macrophages, α4β7+ T cells are accumulated in NASH liver, which induce liver inflammation and fibrosis. αE+ cDC1 and β7+ TH17 are employed to reduce metabolic disorders and steatosis in obese mice. Activated integrin α9β1 is endocytosed by hepatocytes and secreted in the form of extracellular vesicles (EVs), which are further captured by monocytes. Captured integrin α9β1 mediates monocyte adhesion to LSECs by binding to VCAM-1, which accelerates liver fibrosis. αV integrins are the regulators of fibrosis in HSCs and LSECs, making them therapeutic targets in NASH. Additionally, integrin α8β1 promotes liver fibrosis by activating TGF-β in HSCs.

Recently, emerging evidence has suggested that the hepatic microenvironment consists of various types of cells and involves intercellular crosstalk [69]. The pivotal role of integrins in cell-cell communication makes them promising therapeutic targets. Guo *et al*. [70] reported that integrin α9β1 established communication among hepatocytes, monocytes and LSECs. Extracellular vesicles enriched with integrin α9β1, which are derived from lipotoxic hepatocytes, mediate monocyte adhesion to LSECs ( Figure 5). In a separate study, the loss of integrin β1 in hepatocytes induced liver fibrosis through an increase in TGF-β level [71].

## Integrins and NAFLD-related HCC

NAFLD has emerged as a major risk factor for HCC and is correlated with elevated expression of integrin β1 and activation of its downstream phospho-FAK. Blocking the integrin β1/FAK pathway in liver cancer cells alleviates NAFLD-related HCC in animal models [72]. Among the β1 integrins, the expression of integrin α5β1 is highest in HCC tumors. Fibronectin in fibroblasts is remodeled by upregulated integrin α5β1 in cancer cells, promoting tumor growth and angiogenesis [73]. In addition to β1 integrins, which are crucial for the progression of NAFLD, β4 integrins and other integrins have also been reported to play significant roles in the development of liver cancer [ 13, 74]. For example, research has shown that integrin α6β4 is overexpressed in HCC and promotes metastasis, invasion and the EMT process by conferring anchorage independence through EGFR-dependent FAK/AKT activation [ 75, 76]. The function of β3 integrins in liver cancer remains controversial. Integrin αVβ3 has been reported to facilitate the invasion and metastasis of HCC cells and is overexpressed in HCC tissues [ 77, 78]. However, β3 integrins and their ligands were downregulated in 60% of the HCC samples, as reported by Wu *et al*. [79], suggesting a potential therapeutic approach to restrain the aggressive growth of liver cancer.

## Integrin-targeting Diagnostics and Treatments

The acknowledged role of integrins in tumor development has rendered them promising targets for cancer therapy in recent years. Various integrin antagonists, such as antibodies and synthetic peptides, have demonstrated their efficacy in inhibiting tumor progression in preclinical and clinical research.

### Application of integrins in NAFLD-related diseases

Molecular imaging is a vital component of precision medicine, contributing to early diagnosis, staging, tailored treatment, prognostic evaluation, prognostic evaluation, and monitoring of therapeutic efficacy for life-threatening diseases such as cancer. Polypeptides containing RGD sequences have been used as probes in SPECT/PETCT imaging agents in clinical trials because they primarily target integrin αV, which is overexpressed in tumor neovascular endothelial cells and numerous tumor cells ( Table 1) [17]. As early as 2004, Sipos *et al*. [80] reported the overexpression of integrin αV in gastrointestinal pancreatic cancer. Integrin αVβ6 is strongly expressed in hilar cholangiocarcinoma and intrahepatic cholangiocarcinoma but not in HCC [81], potentially serving as a prospective immunohistochemical marker with specificity in the differential diagnosis of primary liver cancers. In contrast, integrin αVβ3 has been reported to be overexpressed in carcinoma tissue and to mediate the invasion and metastasis of HCC cells [77]. Zheng *et al*. [82] investigated the feasibility of 99mTc-HYNIC-PEG4-E[PEG4-c(RGDfK)]2 for the detection of HCC in tumor-bearing mice. Integrin αVβ3 has garnered much attention in the clinical diagnosis of solid tumors, and improving its accuracy in the diagnosis of liver cancer is extremely important. In addition, Lin *et al*. [83] designed an optimized integrin α6-targeted magnetic resonance imaging (MRI) probe called DOTA(Gd)-ANADYWR for mouse HCC MRI. However, whether this integrin can become a diagnostic target in humans remains to be verified.

**Table:** **Table 1** The use of integrins in diagnostic imaging

Disease	Targeted integrin	Source	Drug name	Diagnosis method	Time (first posted)	Phase
Malignant solid tumors	αVβ3	CTR20222903	^68^Ga-HX01	PET/CT	2022-11-10	Phase I
Malignant solid tumors	αVβ6	ChiCTR2200066067/NCT05835570	^68^Ga-Trivehexin	PET/CT	2022-11-23	Unknown
Solid tumors	αVβ3/αVβ5	NCT04712721	^68^Ga-FF58	PET/CT	2021-10-14	Phase I
Solid tumors	αVβ6	NCT04285996	[ ^18^F]-FBA-A20FMDV2	PET/CT	2016-03	Unknown
Steatohepatitis	αVβ6	NCT04063826	[ ^18^F]-FBA-A20FMDV2	PET/CT	2018-04-10	Unknown

Animal experiments have shown that [ ^18^F]-F-FPP-RGD _2_ [84] and [ ^18^F]-Alfatide [85] appear to be promising PET imaging radiotracers for monitoring hepatic integrin αV protein levels and hepatic function in liver fibrotic pathology. A clinical trial involved the utilization of [ ^18^F]-FBA-A20FMDV2 PET to quantify integrin αVβ6 in healthy and fatty liver tissues (NCT04063826). The aforementioned research laid the groundwork for a succession of ongoing clinical applications that utilize [ ^18^F]-FBA-A20FMDV2 as a radioligand in PET/CT studies to identify integrin αVβ6. As a result, [ ^18^F]-FBA-A20FMDV2 can serve as a reversible, specific, and selective PET ligand for αV integrins, as well as an imaging tool applicable to human subjects for monitoring the clinical efficacy of novel therapies in incurable and life-limiting diseases such as liver fibrosis.

### Integrins as targets for inflammatory disease therapeutics

Certain specific leukocyte integrins are activated by inflammatory cytokines during inflammation, thereby encouraging cellular adherence to their receptors and enabling phagocytosis and cytotoxic killing. Many integrins have been designated as potential therapeutic targets for small compounds, peptides, and/or monoclonal antibodies. Currently, therapeutic interventions targeting α4 integrins for the treatment of multiple sclerosis (MS), as well as β7 integrins (α4β7 and αEβ7 integrins), for the management of inflammatory bowel disease (IBD) have been implemented. Many large-scale clinical trials have been conducted to assess the efficacy of etrolizumab (anti-β7) in patients with IBD. Etrolizumab inhibits leukocyte gut homing and retention by blocking α4β7 and αEβ7 integrins, respectively [86]. The efficacy of other anti-integrin β7 therapies in the treatment of colitis, such as abrilumab (anti-α4β7), PN-943 (orally administered and gut-restricted α4β7 antagonist peptide) and AJM300 (orally active small molecule inhibitor of α4), is not known. In patients with type 1 diabetes (T1D), α4β7 integrin assists immune cells in trafficking from the periphery to the target tissue, leading to destruction of islet cells [87]. Vedolizumab directly blocks integrin α4β7 on circulating immune cells, preventing their egress from the blood and relieving T1D. Clinical trials have evaluated the immune effects of vedolizumab plus anti-TNF pretreatment in T1D, which blocks TNF-α signaling and its related expression of the α4β7 ligand MAdCAM-1 in pancreatic endothelial cells (NCT05281614).

The integrins αVβ3 and α5β1 are involved in the pathogenesis of rheumatoid arthritis (RA). α5β1 and αVβ3 are highly expressed in fibroblasts during inflammation, which is concomitant with an increase in the release of proinflammatory mediators, such as matrix metalloproteinases (MMPs) and osteoclast activators, which are receptor activators of NF-κB ligands. The adhesion of lymphocytes expressing α4β1 or α5β1 to ECM ligands induces the expression of inflammatory factors that promote the proliferation and survival of synoviocytes and chondrocytes, which results in hyperplasia of synovial tissue and destruction of bone and cartilage [88]. A small-molecule αVβ3 antagonist has been reported to be efficacious in a rabbit model of RA [89]. Etaracizumab is recognized as a humanized anti-αVβ3 monoclonal antibody and has entered phase II clinical trials as a medication for RA treatment. Nevertheless, the phase II trial for the treatment of RA in humans has been terminated as a result of severe observed adverse effects, including myocardial infarction, atrial fibrillation and thromboembolic events. Clinical trials targeting αVβ3 with other antibodies or small molecules for RA are currently underway ( Table 2) [88].

**Table:** **Table 2** Integrin-targeting therapies in clinical trials of inflammatory diseases

Disease	Targeted integrin	Source	Drug name	Drug type	Time (first posted)	Phase
Ulcerative colitis and Crohn’s disease	α4β7	BLA761133	Vedolizumab	Monoclonal antibody	2014-05	FDA approved
Type 1 diabetes	α4β7	NCT05281614	Vedolizumab	Monoclonal antibody	2022-09-21	Phase I
Ulcerative colitis	α4β7	NCT04504383	PN-943	Peptide	2020-08-05	Phase II
Multiple sclerosis and Crohn’s disease	α4	BLA125104	Natalizumab	Monoclonal antibody	2004-09	FDA approved
Psoriasis	αVβ3	NCT00192517	MEDI-522	Monoclonal antibody	2003-12	Phase II
Rheumatoid arthritis	αVβ3	NCT00069017	MEDI-522	Monoclonal antibody	2003-09	Phase II
NASH and idiopathic pulmonary fibrosis	αvβ1, αvβ3 and αvβ6	NCT03949530	IDL-2965	Small molecule	2019-04-16	Phase I
Osteoarthritis	α10β1	NCT05344157	XSTEM-OA	Mesenchymal stem cells	2022-06-22	Phase I/II

While the majority of integrin therapeutic antagonists demonstrate better bioavailability during clinical trials focused on inflammatory diseases, their efficacy in treating NAFLD remains undetermined. Consequently, integrins, such as integrin α4β7, remain prospective therapeutic targets for the management of NAFLD, and further investigations need to be conducted in this regard.

### Integrins as targets for liver cancer therapeutics

Numerous integrins contribute to cell-ECM and cell-cell interactions, which have also been linked to fibrosis, inflammation, thrombosis, and tumor metastasis. Since many solid tumors originate from epithelial cells, the integrins expressed by epithelial cells, such as α2β1, α3β1, α6β1, α6β4, and αVβ5, are typically preserved within the tumor. Although their primary function is to facilitate the adhesion of epithelial cells to the basement membrane, these integrins may also play a role in the migration, proliferation and survival of tumor cells. Notably, the levels of integrins α5β1, αVβ3 and αVβ6 are often negligible or undetectable in the majority of adult epithelia but can be highly upregulated in certain malignancies. Considering the extensive research on tumors, these integrins may emerge as promising targets for cancer therapy. Furthermore, certain integrin antagonists have been effectively utilized in the treatment of cancer in clinical settings.

A phase I clinical trial demonstrated the safety and tolerability of intetumumab, a protein that binds with high affinity to multiple αV integrins. Patients whose tumor cells expressed αVβ3 integrin exhibited a prolonged response to intetumumab, whereas those whose tumors expressed αVβ1 integrin only demonstrated a partial response [90]. Nonetheless, the development of this drug was halted during its phase II clinical study for the treatment of melanoma and prostate cancer. Volociximab is a chimeric monoclonal antibody designed to specifically target α5β1 integrin and disrupt its interaction with fibronectin. In phase I and II clinical trials, this anti-α5β1 monoclonal antibody has been evaluated both as a single therapy and in combination with classical drugs such as carboplatin and paclitaxel to treat distinct tumor types, including metastatic melanoma and advanced non-small cell lung cancer. After six cycles of treatment, the preliminary findings indicated a median progression-free survival increase of 6.3 months and reduced concentrations of potential biomarkers associated with angiogenesis or metastasis [91].

Several recent studies have indicated that integrins are involved in cancer development. However, the efficacy of integrin-based therapy for liver cancer is limited to animal experiments. Observing the performance of these anti-integrin agents in HCC clinical trials and investigating how their efficacy might be optimized in conjunction with additional therapy options would be interesting ( Table 3).

**Table:** **Table 3** Clinical trials for the assessment of integrin-targeting therapeutics in liver cancer

Disease	Targeted integrin	Source	Drug name	Drug type	Time (first posted)	Phase
Advanced non-hematologic malignancies	α5β1	NCT00915278	PF-04605412	Monoclonal antibody	2009-09	Phase I
Advanced solid tumors and glioblastoma multiforme	αVβ6	NCT01122888	EMD121974	Cyclic peptide	2009-12	Phase I
Pancreatic cancer and solid tumor malignancies	αVβ3	NCT05085548	ProAgio	Protein drug	2021-10-29	Phase I
Advanced colorectal cancer	αVβ3	NCT00027729	MEDI-522	Monoclonal antibody	2001-06	Phase I/II
Refractory prostate cancer	αV	NCT00537381	Intetumumab (CNTO 95)	Monoclonal antibody	2007-05	Phase II
Melanoma	αV	NCT00246012	Intetumumab (CNTO 95)	Monoclonal antibody	2005-05	Phase I/II
Metastatic colorectal cancer	αV	NCT03688230	Abituzumab (EMD525797)	Monoclonal antibody	2019-04	Phase II
Metastatic melanoma	α5β1	NCT00099970	Volociximab (M200)	Monoclonal antibody	2004-12	Phase II

## Challenges and Prospects

The new generation of imaging agents that target integrins offers new promise for diagnosing liver fibrosis and solid tumors. Although αVβ3 is considered a promising diagnostic target for tumors and fibrosis, its expression levels remain nonnegligible in certain organs, leading to substantial background uptake and unwanted organ doses [92]. Therefore, αVβ3-targeted radiopharmaceuticals have not yet been developed for routine clinical diagnosis of cancer and fibrosis. Additionally, αVβ3 integrin has been found on other cells, such as macrophages [93]. Further work in this field is expected to expand the scope of integrin-targeted optical imaging, including improving optical probes and discovering new ligands targeting integrins.

Owing to the characteristic features and complex molecular mechanisms of integrins, progress in drug discovery targeting integrins has not been ideal. An important lesson from past integrin drug development efforts is that the success of integrin drug discovery depends on unmet clinical needs and a deep understanding of the fundamental mechanisms of cell adhesion. Currently, the mechanism of most antibody drugs, peptides or small molecule antagonists that target integrins lies in blocking the binding between biological ligands and integrins. Because integrins undergo significant conformational changes during activation, inhibitors that target the activation process have been proposed as drug targets [26]. Although conformer-specific inhibitors have been developed by the pharmaceutical industry, none have entered the market. This may be related to the limited specificity of these conformer-related inhibitors, as well as the unexpected systemic toxicity caused by the inappropriate binding of these antagonists inducing conformational changes in integrins. Additionally, integrin drugs that are administered orally are still under development or are undergoing clinical trials [94]. Factors contributing to the lack of oral small molecules that target integrins include mainly the polar pharmacophores of these molecules and the complex pharmacology of the target pathway. An in-depth understanding of the pathogenic mechanisms of NAFLD provides hope for the treatment of NAFLD-NASH. Integrin αV is considered a crucial target for treating fibrosis. The most advanced integrin-targeted therapy for NASH is the selective αvβ1 inhibitor PLN-1474 [26]. However, owing to strategic adjustments, Novartis AG terminated the collaboration and development of the integrin αVβ1 inhibitor PLN-1474 for NASH treatment in 2023. IDL-2965 is an oral integrin αV antagonist that has been investigated as a potential treatment for NASH. Unfortunately, owing to the challenges associated with the COVID-19 pandemic and newly emerging nonclinical data, the NCT03949530 study evaluating IDL-2965 was terminated prematurely [41].

Among the FDA-approved integrin drugs, natalizumab is used to treat MS and Crohn’s disease. Patients receiving natalizumab therapy experienced the unexpected development of progressive multifocal leukoencephalopathy (PML), leading to the withdrawal of this drug from the market in 2005. However, owing to its significant benefits, natalizumab returned to the market in 2006. Owing to the risk of PML associated with the use of natalizumab, vedolizumab has effectively replaced it in clinical practice for the treatment of ulcerative colitis and Crohn’s disease. Moreover, on the basis of MAdCAM-1 expression level, potential new indications for vedolizumab include chronic liver diseases [95]. Comprehensive safety data from over 4000 patient-years of vedolizumab exposure in six clinical trials indicate good long-term tolerability and acceptable safety for patients receiving vedolizumab treatment [96].

In summary, the global incidence of NAFLD/NASH is increasing. However, the current lack of effective treatments for NASH persists, with several others ( *e*. *g*., elafibranor, seladelpar, emricasan, selonsertib and elobixibat) having already been deemed ineffective [97]. Consequently, there is an urgent imperative for the development of effective treatments to mitigate the increasing prevalence and mortality associated with NAFLD. According to reported findings, aberrant expression, activation, and signaling pathways, in alignment with the multifaceted functions of integrins, are involved in almost every stage of NAFLD development, including NAFLD, NASH, fibrosis, cirrhosis and HCC. The distinctive and intricate role of integrins could provide potential therapeutic targets for liver diseases. Animal models of NAFLD and NASH have demonstrated that inhibitors targeting HSC- and LSEC-expressing integrins, such as αVβ1 and αVβ6, can effectively attenuate lipid accumulation and fibrosis. Unfortunately, satisfactory outcomes have yet to materialize in clinical trials, underscoring the necessity for additional data derived from human disease samples. Cell adhesion plays a vital role in restricting the excessive activity of immune cells toward inflammatory tissues. Currently, integrin αM ^+^ macrophages, αE ^+^ dendritic cells, and α4β7 ^+^ T cells are reported to be involved in the progression of NAFLD. Although monoclonal antibodies have been utilized to prevent leukocyte adhesion, their practical implementation has frequently been unsatisfactory as a result of undesirable side effects [98]. We posit that sustained efforts to enhance our knowledge of the function of integrins in NAFLD will pave the way for the development of more innovative targeted approaches and usher in a renaissance in this area.
